# Revealing the Potential Application of EC-Synthetic Retinoid Analogues in Anticancer Therapy

**DOI:** 10.3390/molecules26020506

**Published:** 2021-01-19

**Authors:** Mohamed R. Abdelaal, Sameh H. Soror, Mohamed R. Elnagar, Hesham Haffez

**Affiliations:** 1 Biochemistry and Molecular Biology Department, Faculty of Pharmacy, Helwan University, Cairo 11795, Egypt; mohamed_abdelaal@pharm.helwan.edu.eg (M.R.A.); sameh_soror@pharm.helwan.edu.eg (S.H.S.); 2 Center of Scientific Excellence “Helwan Structural Biology Research, (HSBR)”, Helwan University, Cairo 11795, Egypt; 3 Department of Pharmacology and Toxicology, Faculty of Pharmacy, Al-Azhar University, Cairo 11823, Egypt; mohamed.r.elnagar@azhar.edu.eg

**Keywords:** anticancer, apoptosis, Caco-2, EC19, EC23, metastasis, retinoic-acid receptors

## Abstract

(1) Background and Aim: All-*trans* retinoic acid (ATRA) induces differentiation and inhibits growth of many cancer cells. However, resistance develops rapidly prompting the urgent need for new synthetic and potent derivatives. EC19 and EC23 are two synthetic retinoids with potent stem cell neuro-differentiation activity. Here, these compounds were screened for their in vitro antiproliferative and cytotoxic activity using an array of different cancer cell lines. (2) Methods: MTT (3-(4,5-dimethylthiazol-2-yl)-2,5-diphenyltetrazolium bromide) assay, AV/PI (annexin V-fluorescein isothiocyanate (FITC)/propidium iodide (PI)), cell cycle analysis, immunocytochemistry, gene expression analysis, Western blotting, measurement of glutamate and total antioxidant concentrations were recruited. (3) Results: HepG2, Caco-2, and MCF-7 were the most sensitive cell lines; HepG2 (ATRA; 36.2, EC19; 42.2 and EC23; 0.74 µM), Caco-2 (ATRA; 58.0, EC19; 10.8 and EC23; 14.7 µM) and MCF-7 (ATRA; 99.0, EC19; 9.4 and EC23; 5.56 µM). Caco-2 cells were selected for further biochemical investigations. Isobologram analysis revealed the combined synergistic effects with 5-fluorouracil with substantial reduction in IC_50_. All retinoids induced apoptosis but EC19 had higher potency, with significant cell cycle arrest at subG_0_-G_1_, -S and G_2_/M phases, than ATRA and EC23. Moreover, EC19 reduced cellular metastasis in a transwell invasion assay due to overexpression of E-cadherin, retinoic acid-induced 2 (*RAI2*) and Werner (*WRN*) genes. (4) Conclusion: The present study suggests that EC-synthetic retinoids, particularly EC19, can be effective, alone or in combinations, for potential anticancer activity to colorectal cancer. Further in vivo studies are recommended to pave the way for clinical applications.

## 1. Introduction

Retinoids are naturally occurring structural and functional analogues of vitamin A that play a vital role in mammalian physiology during infancy and adulthood [1]. They influence many intracellular pathways by activating a number of trans-acting, DNA-binding receptors. This family of nuclear receptors includes retinoic acid receptors (RARs) and retinoid X receptors (RXRs). Each receptor has 3 isotypes: RA(X)Rα, RA(X)Rβ and RA(X)Rγ [2]. Alternative splicing and differential promoter activation generate several isoforms from each isotype [3]. RARs are naturally targeted by all-*trans*-retinoic acid (ATRA), the main intracellular metabolite of vitamin A [4]. However, other natural analogues such as 9-*cis*-RA and 13-*cis*-RA mainly activate RXRs [5]. Retinoids are important for diverse biological processes such as embryonic morphogenesis, cell growth, and hematopoiesis in vitro and in vivo [6]. Also, retinoic acid (RA) has a protective action on the epithelium through preventing ultraviolet-induced damage to the skin following exposure to ultraviolet radiation [7]. On the cellular level, retinoids are involved in diverse biological activities such as cellular growth and cohesion in stem cells [8], immunomodulatory effects [9] and growth inhibition particularly in cancer cells [10,11]. Standard chemotherapeutic treatments against cancer are necessary for surgery inappropriate patients and after surgical resection to improve prospects for survival and surgery outcomes [12]. However, the issue of multidrug resistance (MDR) is a major obstacle to obtain the desired results [13], making the discovery of novel tumor inhibitors necessary for effective therapeutic strategies that help solving the problem. However, this process is durable, costly and most of these new drugs fail in the clinic because of undesired side effects and toxicity [14]. As a result, there is an urgent need for repurposing effective synthetic derivatives to alleviate the emerging resistance and provide higher stability under different treatment environments. In the last few decades, retinoids attracted great attention as promising drugs for cancer prevention and treatment [15]. ATRA has been clinically tested in acute promyelocytic leukemia [16,17], prostate cancer [18,19], and other types of cancer [20]. However, ATRA resistance rapidly emerged after starting the treatment [18,21]. Moreover, it undergoes photoisomerization and degradation in solution into a mixture of retinoic acid isomers [22,23] with different mechanisms of action [24,25]. This may lead to inconsistency with ATRA dosing in clinical settings.

**Figure 1:**
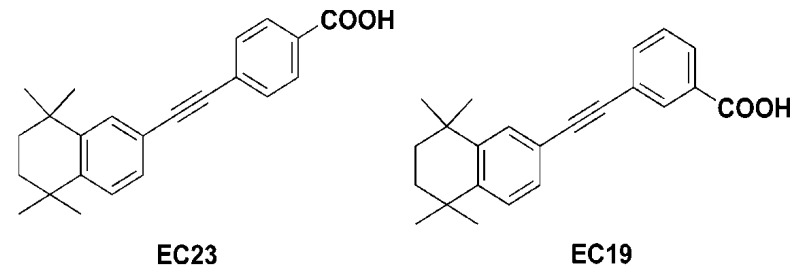
Structures of EC-synthetic retinoic acid analogues [26].

Therefore, on the scaffold of ATRA, two chemical derivatives called EC-synthetic retinoids have been synthesized by Whiting’s group. The *para*-isomer,4-(5,5,8,8-tetramethyl-5,6,7,8-tetrahydronaphthalen-2-ylethynyl) benzoic acid, is often called EC23 while the *meta*-isomer,3-(5,5,8,8-tetramethyl-5,6,7,8-tetrahydronaphthalen-2-ylethynyl) benzoic acid, is called EC19 (Figure 1) [26]. The two analogues showed higher physicochemical stability for extended periods under the laboratory environment [26]. Additionally, intensive research on cellular [27], biochemical [28] and molecular levels [28] was performed at different time scales to probe the relationship between the structures and functions of EC23 and EC19. In vivo results showed that EC23 induces increased maturation and stabilization of the axonal cytoskeleton [29] and developing limb bud [30]. In vitro data support the previous observation that EC23 is a potent inducer of pluripotent stem cell differentiation (TERA2.cl.SP12) and neuroblastoma (SHSY5Y) with the same mechanism as ATRA [27,28].

Although intensive research work was done by our research group on EC-synthetic analogues for their potential application in neuroscience as inducers of neuro-differentiation, there is no prior information about the efficiency of these analogues compared to ATRA for potential growth inhibition in cancer. This may be a good approach to aid ligand design and for repurposing of EC-synthetic retinoids as anticancer ligands benefitting from their activity on cell differentiation. Therefore, we seek to determine their antiproliferative potency through RARs and other pathways such as apoptosis, drug resistance and cell metastasis, and thus understand determinants responsible for the biological potency of these tested retinoids.

## 2. Results

### 2.1. EC19 and EC23 Induce Cell Death In Vitro

The antiproliferative effect of EC19 and EC23, compared to ATRA as a positive control retinoid, was assessed on cultured cancer cells using the MTT assay. As shown in (Figure S1), different sigmoidal curves were constructed for each specific cell line with retinoids for calculation of IC_50_. The dynamic dose concentrations were chosen to cover the bend points of the sigmoidal curves and cover the expected change in IC_50_ values. A constant dilution factor of 10× was chosen to minimize the variability with logarithmic scale dilution change, as previously documented [31,32]. The magnitude of reduction in cell growth was dose-dependent and varied for each retinoid molecule markedly among different cell lines, in comparison to ATRA and negative controls. Table 1 shows the different IC_50_ values for EC-synthetic analogues in comparison to ATRA. For hepatocellular carcinoma cell lines, HepG2 showed high sensitivity to retinoids at IC_50_ doses lower than 100 µM compared to Huh7 cells. EC23 showed the lowest IC_50_ in HepG2 cells (0.74 ± 0.001 µM) compared to EC19 and ATRA (IC_50_ values; 42.2 ± 0.92 and 36.2 ± 1.9 µM, respectively). For colorectal carcinoma (CRC), Caco-2 was the most sensitive cell line to different retinoids with the highest antiproliferation potency for EC19 (IC_50_; 10.8 ± 0.1 µM), followed by EC23 (IC_50_; 14.7 ± 0.73 µM) compared to ATRA (IC_50_; 58.0 ± 1.0 µM). HCT-116 and HT-29 exhibited variable sensitivity to tested retinoids with only EC23 potent in HCT-116 (IC_50_; 44.46 ± 1.1 µM) and ATRA potent in HT-29 (IC_50_; 0.002 ± 0.01 µM). For breast cancer cells, MCF-7 was the most sensitive cell line to retinoids where EC23 was the most potent antiproliferative compound (IC_50_; 5.56 ± 0.01 µM), followed by EC19 (IC_50_; 9.4 ± 0.13 µM) and ATRA (IC_50_; 99 ± 0.26 µM). MDA-MB 231 was resistant to all retinoids with IC_50_ > 100 µM. For prostate and lung cancers, DU145 was the only cell line sensitive to EC19 (IC_50_; 86.9 ± 2.0 µM) while A549 was sensitive only to ATRA (IC_50_; 84.7 ± 3.2 µM). WI-38 and Vero cell lines were recruited for screening the cytotoxicity of these retinoids against normal cells. EC23 showed the most cytotoxic effect to both normal cell lines (WI-38; IC_50_ = 8.01 ± 0.13 and Vero; IC_50_ = 35.23 ± 1.02 µM). EC19 and ATRA showed no cytotoxicity to Vero cells and moderate cytotoxicity to WI-38 (IC_50_ values; 48.4 ± 1.2 and 34.0 ± 1.1 µM, respectively). Therefore, WI-38 was selected as the normal cell line model of human origin to assess the selectivity and cytotoxicity of the tested retinoids compared with other cancer cell lines. Data shown in Table 1 demonstrate that EC19 had selectivity index (SI) > 1 for three cancer cell lines (HepG2, Caco-2 and MCF-7). EC23 had SI > 1 for two cancer cell lines (HepG2 and MCF-7). ATRA was shown to have SI > 1 against only one cancer cell line (MCF-7). It is well known from the literature that anticancer agents with greater SI value (specifically when SI > 1) are more selective and safer for future in vivo studies [33,34,35]. The SI data shown in Table 1 indicate that EC19 and EC23 have a relatively safe effect on Caco-2 and HepG2, respectively. Therefore, due to the comparable IC_50_ of EC19 and EC23 to the Caco-2 cell line versus ATRA, this cell line will be selected for further biochemical and mechanistic investigations to understand their potency and mechanism of antiproliferative activity.

### 2.2. Synergistic Effect of Retinoids and 5-Fluorouracil (5-FU) in Caco-2 Cells

**Figure 2:**
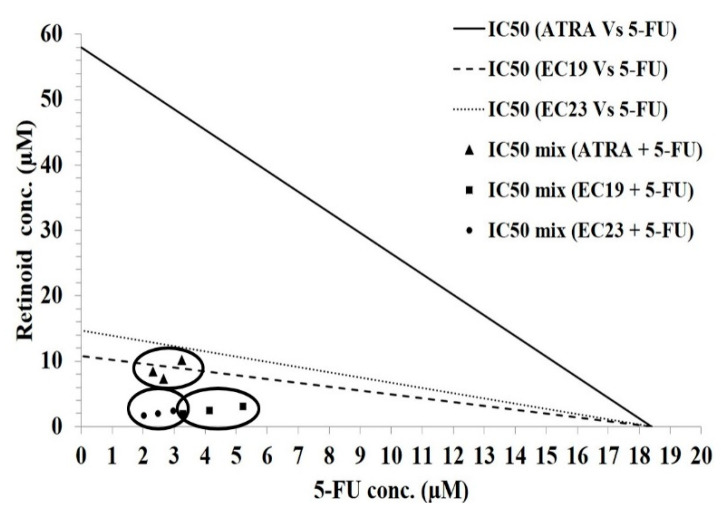
Isobologram analysis applied to Caco-2 cells co-treated with 5-FU in addition to ATRA, EC19, or EC23 for 24 h. The antagonistic (upper), the synergistic (lower), and the additivity (on the line) regions are shown. The analysis indicates synergism at the IC_50_ level. Data points in circles represent the mean of 3 independent replicates.

The effect of the combined administration of ATRA, EC19 or EC23 with the standard genotoxic agent, 5-fluorouracil (5-FU), was assessed on the Caco-2 cell line using isobologram analysis. First, the non-linear regression model, fitted from MTT assay data, revealed that single treatment with 5-FU yields an IC_50_ value of 18.4 ± 0.6 µM. Next, IC_50_ values of ATRA, EC19 and EC23 were combined with IC_50_ of 5-FU (with fixed ratios) onto the cells and an exaggerated response was observed. Table 2 and Figure 2 show the combined IC_50_ values for the mixture containing ATRA, EC19 or EC23, plus 5-FU as 8.7 ± 0.9, 2.47 ± 0.33, and 2 ± 0.22 µM, respectively. Table 2 shows the combination indices (CI) for the mixtures of ATRA, EC19 and EC23 with 5-FU as 0.29 ± 0.03, 0.46 ± 0.06 and 0.27 ± 0.03, respectively. Moreover, the significant reduction in IC_50_ was reflected in high dose reduction index (DRI) values (ranging from 4.5 to 7.5), highlighting the strong reduction in doses needed to achieve the same level of cytotoxicity (Table 2). These results suggest that an augmentation in the anticancer potency of the standard chemotherapeutic agent, 5-FU, can be induced by the combination with the EC-synthetic retinoids in Caco-2 cells. In addition, the IC_50_ doses required for the induction of growth inhibition were reduced by fractions of IC_50_ (×IC_50_) to minimize the cytotoxicity effect that may occur at high doses in non-cancerous neighboring cells.

### 2.3. Apoptotic Effects of Retinoids in Caco-2 Cells

Propidium iodide (PI) is commonly used in conjunction with Annexin-V to discriminate viable cells from those undergoing early or late apoptosis, or necrosis [36]. The annexin V-fluorescein isothiocyanate (FITC)/PI staining (AV/PI) showed Caco-2 cell distribution within the four different quadrants (C^−−^, C^−+^, C^+−^, C^++^) of the 2D plot. In the untreated cells (negative control), there was minimal cell distribution in C^−+^, C^+−^, C^++^ indicating a very low number of necrotic, early, and late apoptotic cells, respectively. Treatment of Caco-2 cells with the IC_50_ concentration of retinoids for 12 and 24 h showed variable apoptotic effects on Caco-2 cells.

After 12 h of treatment, the percentage of viable cells increased with EC19 and EC23 compared to ATRA and negative control (93.65 ± 14.0 and 91.61 ± 13.7, respectively, compared to ATRA; 87.99 ± 15.0 and negative control; 80.30 ± 7.9). Also, there was no significant effect on any of the other apoptotic phases. After 24 h of treatment, there was a significant reduction in the percentage of viable cells with higher potency for EC19 followed by EC23 and ATRA (24.5 ± 11.8, 34.4 ± 18.3 and 52.4 ± 11.5, respectively). Moreover, there was a significant increase in the percentage of early apoptotic cells with the same order of potency as previously mentioned (EC19; 75.3 ± 13.8, EC23; 65.4 ± 18.4 and ATRA; 47.4 ± 2.5) (Table 3). In addition, the significant induction of apoptosis after 24 h was confirmed through the calculation of apoptotic index (AI) which was significantly higher for EC23 and EC19 followed by ATRA compared to the negative control (89, 84 and 63 compared to control; 18). There was no increase in AI after 12 h of treatment.

### 2.4. Synthetic Retinoids Induce Cell Cycle Arrest and Reduce Cellular Proliferation

**Figure 3:**
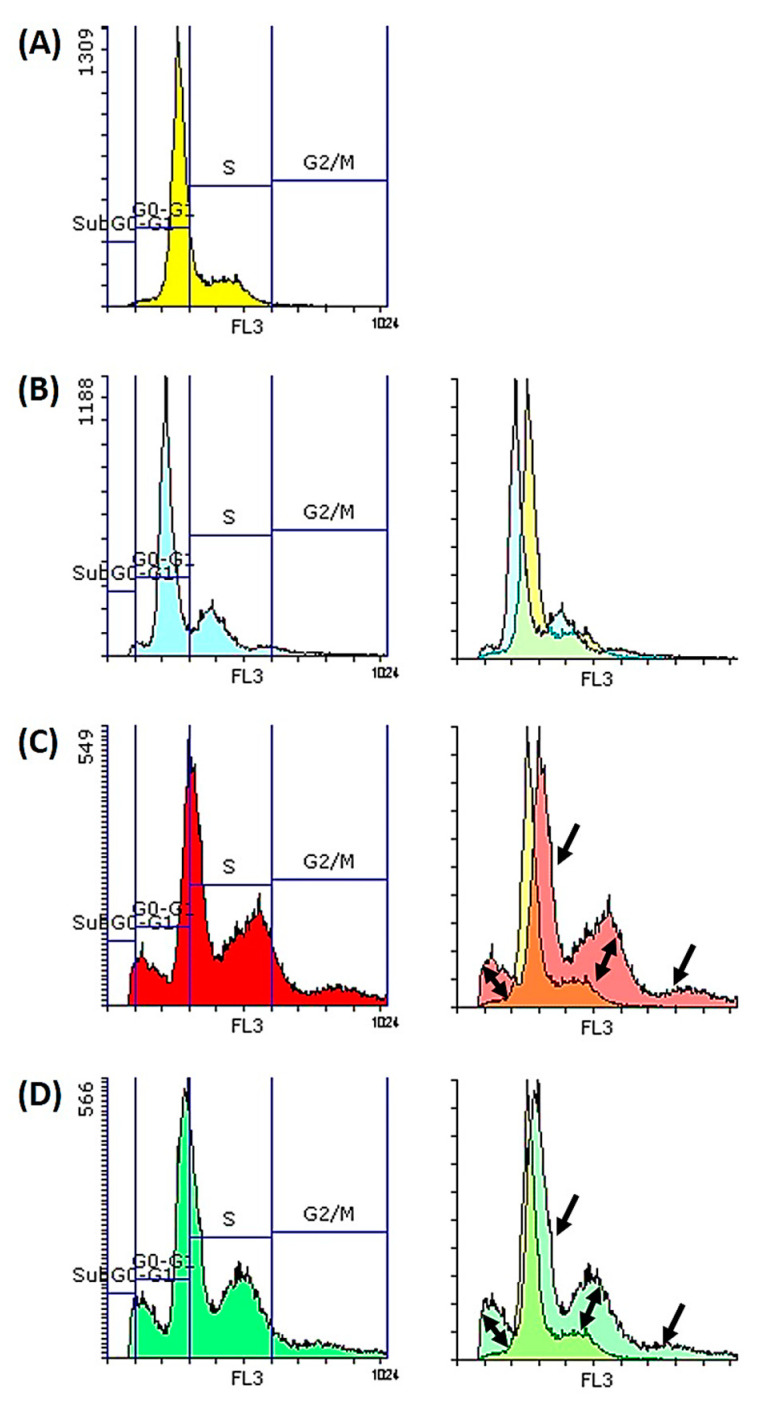
Cell cycle analysis (DNA analysis) of Caco-2 cells treated for 24 h with IC_50_ of retinoids. The histograms of (**A**) negative control exposed to 0.1% DMSO solvent (yellow histogram), (**B**) ATRA (blue histogram), (**C**) EC19 (red histogram), and (**D**) EC23 (green histogram) are shown, together with the overlay histograms of each synthetic retinoid compared to the solvent control. Regions of cell cycle arrest are indicated with black arrows reflecting the early apoptosis compared to control.

Cell cycle analysis was used for the assessment of DNA content in Caco-2 cells after treatment with retinoids for both 12 and 24 h. The percentage of treated cells in all cell cycle phases (G_0_-G_1_, S and G_2_/M) is presented in Table 4. After 12 h of treatment, ATRA and EC19 were able to slightly induce the accumulation of dividing cells in both S- (57.5 ± 8.1 and 51.5 ± 7.2, respectively) and G_2_/M (16.3 ± 1.9 and 9.1 ± 1.3, respectively) phases compared to control cells. EC23 was able to slightly induce the accumulation of cells at the G2/M phase only (6.8 ± 0.8) compared to control. After 24 h of treatment, ATRA was able to induce cell cycle arrest and accumulation of cells at subG_0_-G_1_ and G_2_/M phases (1.6 ± 0.19 and 31.8 ± 4.8, respectively). EC19 and EC23 were able to induce cell cycle arrest at three different levels including subG_0_-G_1_, S- and G_2_/M phases as shown in Table 4; the % G_2_M/G_0_-G_1_ ratio was elevated for all three retinoids after 24-h treatment compared to that of the negative control. Overlaying the different histograms in Figure 3 of the control (yellow color) with that of the different retinoids showed the regions of the cell cycle arrest and confirmed the retrieved data.

### 2.5. Effect of ATRA and Synthetic Retinoids on Cell Invasion

**Figure 4:**
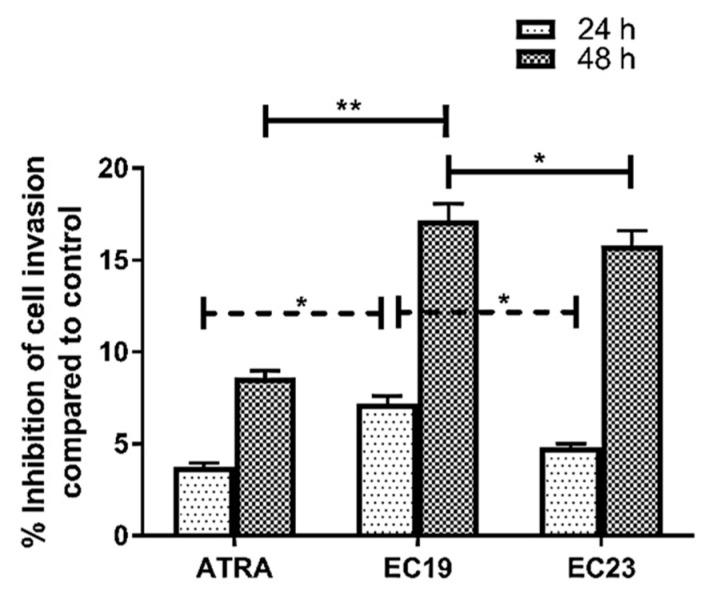
The percentage inhibition of Caco-2 cell invasion after the treatment with retinoids in comparison to the solvent control. The stain of invasive cells that traversed the membrane was extracted using the kit’s extraction buffer and quantified at 490 nm using a microplate reader. The percentage reduction in absorbance (and thus invasion) by retinoids was calculated, in comparison to the DMSO-treated control, and plotted as mean ± SEM of three independent experiments. The most significant reduction was observed with EC19 after 24 h and 48 h compared to ATRA and EC23. The significance in *p*-values for comparison is indicated as * *p <* 0.05, ** *p* < 0.01.

**Figure 5:**
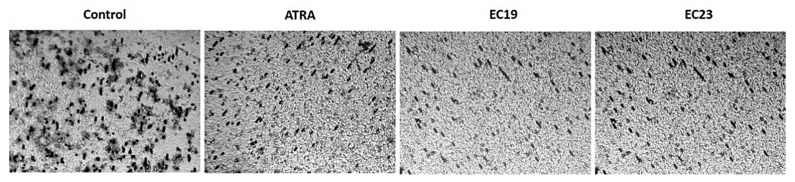
Transwell invasion assay shows the pattern of penetration of Caco-2 cells through the collagen matrix basement membrane. Cells were allowed to invade towards 10% fetal bovine serum for 48 h in the absence or presence of IC_50_ dose of ATRA and EC-synthetic retinoids. The cells penetrating the membrane were stained with violet dye.

The principle for this assay depends on measuring the color intensity of deeply stained penetrated cells as a function of metastasis. Figure 4 shows the effect of ATRA, EC19 and EC23 at IC_50_ concentration after 24 and 48 h of treatment on Caco-2 cell invasion through the polycarbonate filter coated with collagen matrix (i.e., Transwell chamber). When Caco-2 cells were treated with IC_50_ of ATRA or EC-synthetic retinoids for 24 h, there was obvious inhibition in Caco-2 cell invasion compared to control (ATRA (3.8%), EC19 (7.2%), and EC23 (4.8%)). As the time of incubation reached 48 h, the percentage inhibition of Caco-2 cell invasion was increased by the effect of ATRA (8.6%), EC19 (17.2%) and EC23 (15.8%). Also, there was obvious significant reduction in percentage of penetrated cells treated with EC19 after 24 and 48 h compared to both ATRA and EC23 (Figure 4). In the negative control chamber, cells that were stained deep violet were those that successfully invaded the lower surface of the filter as shown in Figure 5. The extent of penetration through the collagen matrix basement membrane by Caco-2 cells was decreased (indicated by the observable reduced violet dots of cells) upon treatment with synthetic retinoids (Figure 5).

### 2.6. EC19 Reduced Intracellular Glutamate in Caco-2 Cells

**Figure 6:**
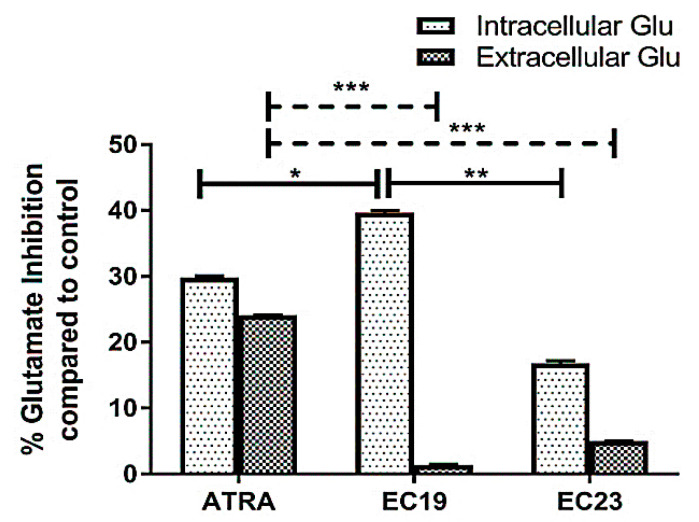
The percentages of both intracellular and extracellular glutamate inhibition. The reduction in glutamate levels was calculated after the manipulation of Caco-2 cells with IC_50_ doses of ATRA and each EC-synthetic analogue. Data are presented as mean ± SEM of three-independent experiments. Significant inhibition of intracellular glutamate with EC19-treated cells was observed compared to ATRA and EC23 treatments. Also, significant inhibition in extracellular secreted glutamate was observed with ATRA compared to both EC19 and EC23. The significance in *p*-values for comparisons is indicated as * *p* < 0.05, ** *p* < 0.01 and *** *p* < 0.001.

The intracellular and extracellular glutamate concentrations were measured as a proxy to reflect the overall changes in glutamate metabolism and release and to understand their correlation with the mechanism of EC-synthetic retinoids in cell growth. The cytosolic glutamate level was markedly reduced to 39.7% (EC19), 29.8% (ATRA), and 16.8% (EC23) compared to the untreated control after the manipulation of Caco-2 cells with IC_50_ doses for 24 h (Figure 6). Also, there was significant reduction observed with EC19-treated cells compared to both ATRA- and EC23- treated cells. The released glutamate was also reduced; however, the magnitude of reduction was 24.1% (by ATRA), 5% (by EC23) and 1.4% (by EC19).

### 2.7. EC19 and EC23 Reduce the Antioxidant Capacity of Caco-2 Cells

**Figure 7:**
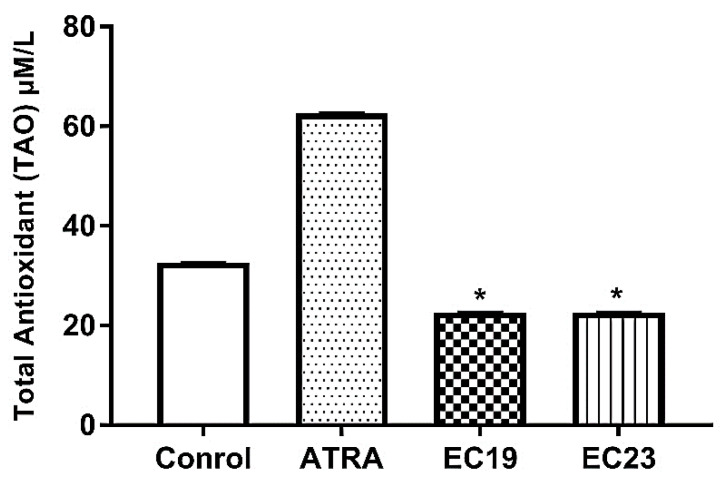
Total antioxidant activity (TAO) of retinoid-treated Caco-2 cells using IC_50_ dose for 24 h. Both EC19 and EC23 induced a significant reduction in the total antioxidants compared to control (treated with 0.1% DMSO). Data is represented as the mean of TOA ± SEM, *n* = 3. * *p* < 0.05.

Total antioxidant capacity (TAC), also called total antioxidant activity (TAO) was determined spectrophotometrically in cell lysates. Figure 7 shows that there were statistically significant differences in TAO values between untreated Caco-2 cells and those treated with IC_50_ concentration of the synthetic retinoids for 24 h. EC19 and EC23 showed a similar reduction in TAO (22.6 ± 0.024 µM/L, *p* < 0.05) in comparison to the solvent control (32.6 ± 0.21 µM/L). In contrast, ATRA induced a significant increase in TAO (62.6 ± 0.15 µM/L, *p* < 0.05) in comparison to the solvent control.

### 2.8. Gene Expression Analysis of Multiple Signaling Genes Affected by ATRA and EC-Synthetic Retinoids Treatments in Caco-2 Cells

It was essential to investigate the possible mechanisms of the anticancer activity and the potency of the synthetic retinoid analogues using some selected signaling genes controlling the carcinogenesis in Caco-2 cells on the mRNA level. Data illustrations are for the IC_50_ doses of different retinoids after 24 h to explain the net response observed in the different aforementioned assays. Gene expression analysis was presented as fold-change; normalized to the control cells treated with 0.1% DMSO solvent. 

#### 2.8.1. Gene Expression Analysis of Retinoic Acid Receptors (RARs)

**Figure 8:**
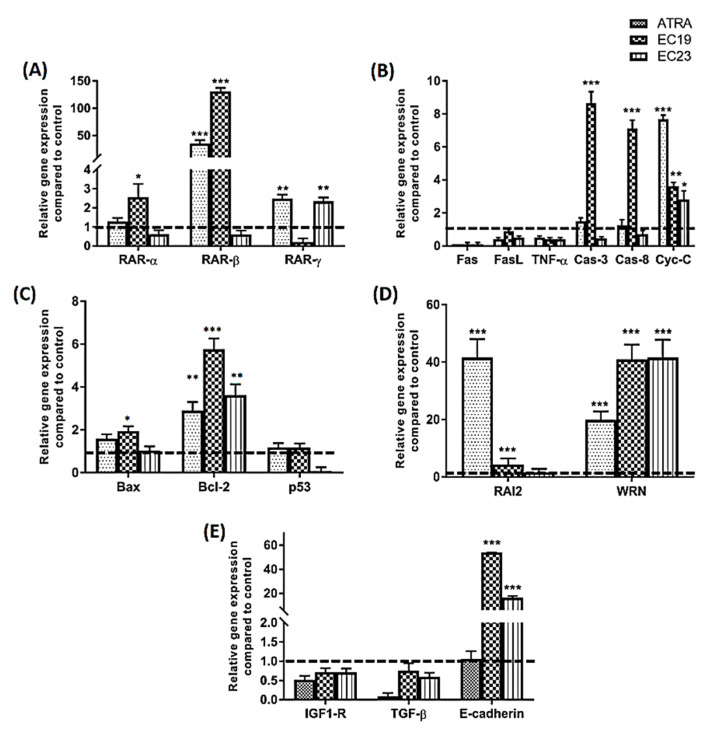
Gene expression analysis for different key genes after treatment of Caco-2 cells with IC_50_ of retinoids for 24 h. Shown are (**A**) RARs (α-, β- and γ-), (**B**) Key regulatory genes for extrinsic and intrinsic apoptosis, (**C**) apoptotic Bax, anti-apoptotic Bcl-2 and p53, (**D**) *RAI2* and *WRN* genes, and (**E**) metastasis and invasion genes (IGF1-R, TGF-β and E-cadherin). The quantification of target mRNA after retinoid treatment was relative to Caco-2 cells incubated with 0.1% DMSO vehicle for 24 h and was normalized to the internal reference gene glyceraldehyde 3-phosphate dehydrogenase (GAPDH) (dashed line at 1). Relative gene expression was calculated by 2^^−ΔΔCt^ method and presented as an average of three independent experiments. The values are considered statistically significant compared to solvent-treated control at * *p* < 0.05, ** *p* < 0.01, *** *p* < 0.001. Data are represented as mean ± SEM, *n* = 3.

RARs are the primary cognate receptors receiving extracellular signals from retinoids including EC19 and EC23 [28]. The induction of RAR-β transcription is well established as a marker of retinoid anticancer response [37]. Therefore, the expression of this gene was tested with respect to specified dose and time treatment, in addition to investigating the retinoid-induced changes in the expression of RAR-α and RAR-γ (Figure 8A). Data obtained after 24 h of treatment with IC_50_ of different retinoids showed that ATRA, the natural RARs ligand, has a 35-fold increase in expression level of RAR-β, a 2.5-fold increase in RAR-γ and no significant effect on RAR-α level. EC23 induced only 2.3-fold increase in RAR-γ with no effect on the expression of other RARs. EC19 was the most potent EC-retinoid where it induced 131-fold increase in RAR-β and 2.6-fold increase in RAR-α without affecting RAR-γ expression.

#### 2.8.2. Gene Expression Analysis of Key Apoptosis-Related Genes

Apoptosis is an essential pathway for retinoids as anticancer agents. Retinoids can induce cell death through either intrinsic or extrinsic pathways. However, there is always an overlap between two signaling pathways dedicated to the activation of the caspase protease family, which is ultimately responsible for elimination of cancer cells. Therefore, it was of value here to investigate the apoptotic mechanism of these synthetic EC-retinoid analogues as apoptotic agents and their potency in comparison to ATRA through measuring the changes on the transcriptomic level. The ability of EC-synthetic analogues to provoke apoptosis in Caco-2 cells was evaluated on the mRNA levels of apoptosis-related genes as shown in Figure 8B. In the present study, Fas, FasL, and TNF-α (death receptors or their ligands) were screened as genes of extrinsic apoptosis in Caco-2 cells [38]. Neither the EC-synthetic retinoids nor ATRA were able to induce the transcription of Fas, FasL or TNF-α. Caspase-3 and -8 (Cas-3 and Cas-8) were also screened for their cascade activation of apoptosis. ATRA induced borderline expression of both caspases with 1.5-fold change (Cas-3) and 1.2-fold change (Cas-8). EC23 did not induce significant expression of any of the two selected caspases. In contrast, EC19 was the most potent retinoid that was able to induce 8.6-fold change (Cas-3) and 7.1-fold change (Cas-8). Additionally, cytochrome-C (Cyt-C) initiates the activation cascade of caspases once it is released into the cytosol [39]. The level of Cyt-C gene expression was screened, and it was found that all retinoids were able to induce over-expression of Cyt-C. However, ATRA showed the highest level of expression with 7.7-fold change, compared to 3.6-fold change (EC19) and 2.8-fold change (EC23). Other important mediators of apoptosis include p53, Bax and its counteracting partner Bcl-2 which are known to control the mechanistic activation of intrinsic apoptosis. Figure 8C shows that ATRA and EC19 had a similar borderline increase in p53 gene expression (1.2-fold change) while EC23 did not induce p53 upregulation. In addition, ATRA and EC19 induced over-expression of the Bax gene with 1.6- and 1.9- fold change, respectively, while EC23 was not able to induce any significant change in Bax gene expression. On the other side, Bcl-2, the anti-apoptotic gene, was still significantly highly expressed after treatment with all retinoids and this was consequently reflected with an overall Bax/Bcl-2 ratio of less than 1.

#### 2.8.3. Gene Expression Analysis of Retinoic Acid-Induced 2 (*RAI2*) Expression in Caco-2 Cells

Retinoic acid-induced 2 (*RAI2*) is a novel tumor suppressor gene whose expression is downregulated in various types of cancer, including breast cancer and CRC [40]. Restoration of *RAI2* expression has been linked to cell apoptosis induction and a good prognosis of colon cancer. Our results show that all retinoids induce upregulation in *RAI2* expression after 24 h with 41.6-fold change (ATRA), 4.4-fold change (EC19) and 1.6-fold change (EC23) (Figure 8D).

#### 2.8.4. Gene Expression Analysis of *WRN* Expression in Caco-2 Cells

Werner (*WRN*) gene is a tumor suppressor gene that was demonstrated to be hypermethylated with reduced mRNA expression and low gene copy number in advanced CRC [41]. Hence, the restoration of *WRN* expression induces tumor-suppressor-like features and inhibits tumor growth and associated carcinogenesis [42]. Our data in Figure 8D showed that all retinoids induced over-expression of *WRN* gene where EC19 and EC23 had similar potency (~40-fold change) compared to ATRA which induced 20-fold change expression of the gene.

#### 2.8.5. Gene Expression of Migration-Related Genes in Caco-2 Cells

The migration and invasion features play vital roles in the development of colon cancer [43]. Some genes are highly modulated and involved in invasion control via epithelial-mesenchymal transition (EMT) responses; including insulin-like growth factor-1 receptor (IGF1-R) [44], transforming growth factor-β1 (TGF-β1) [45]. and E-cadherin [46]. The data presented in Figure 8E depicted that all retinoids were able to significantly reduce the gene expression of IGF1-R and TGF-β1 to the minimal levels. On the other side, recent studies correlate the morphological and phenotypic EMT-like changes with the down-regulation of the epithelial marker E-cadherin [46,47,48]. E-cadherin gene was over-expressed in both EC19 and EC23-treated Caco-2 cells with 54- and 16.3- fold changes, respectively. ATRA did not show any significant increase in the E-cadherin gene expression level (Figure 8E).

### 2.9. Western Blotting Analysis of Key Apoptosis-Related Proteins 

**Figure 9:**
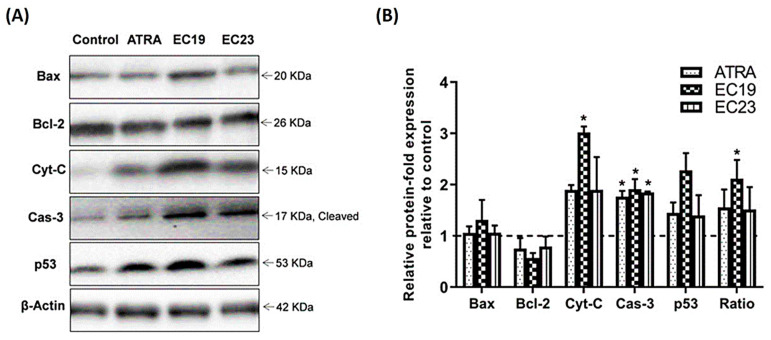
Western blotting analysis of Bax, Bcl-2, Cyt-C, Cas-3 p53 and Ratio (Bax/Bcl-2) in Caco-2 cells. (**A**) Representative immunoblotting images demonstrate the effect of IC_50_ dose of ATRA, EC19, and EC23 on the protein expression levels of Bax, Bcl-2, Cyt-C, cleaved Cas-3, and p53 in Caco-2 cells treated for 24 h. (**B**) Quantification of the tested proteins in Caco-2 cell lysates, with normalization to the β-actin protein (dashed line). The expression of all recorded proteins in the control group is set to (“1”), and all data from three separate experiments are fold change of proteins expression to control (dashed line) and shown as mean ± SEM. * *p* < 0.05 indicates a statistically significant difference from the matching vehicle-treated control group.

To verify the observed effects from gene expression data for the tested compounds on the Caco-2 cell line, the protein levels of Bax, Bcl-2, Cyt-C, Cas-3 and p53 were investigated using Western blotting (Figure 9). While no significant change in the regulation of the pro-apoptotic Bax and the anti-apoptotic Bcl-2 proteins for all the tested compounds, both ATRA and EC19 caused increment in the level of activated Cas-3. Interestingly, the levels of Cyt-C and p53 proteins were significantly increased in Caco-2 cells exposed to EC19 compared to the control cells.

### 2.10. Morphological and Immunocytochemistry Analysis of Caco-2 Cells Treated with Synthetic EC-Retinoids

**Figure 10:**
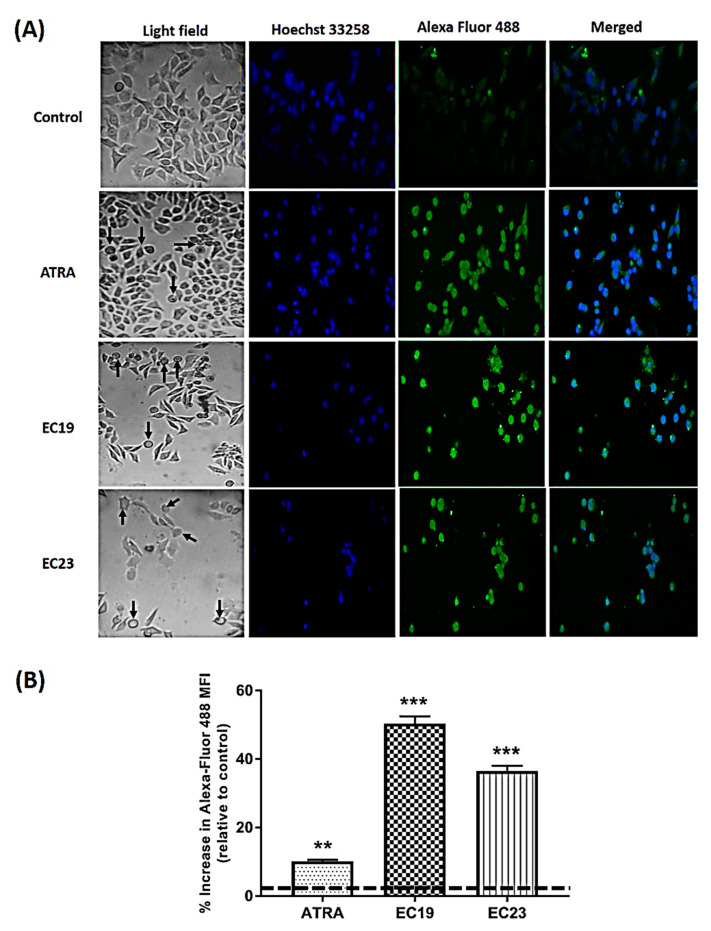
Cell morphology and immunocytochemistry of Caco-2 cells treated with IC_50_ of retinoids for 24 h and stained with anti-Cytochrome-C antibody. (**A**) Control Caco-2 cells treated with 0.1% DMSO was compared to those treated with retinoids under an inverted microscope (OPTECH Biostar IB; magnification, 40×). Dark arrows indicate rounded apoptotic bodies. (**B**) Also, a comparative assessment of anti-Cytochrome-C staining of control cells, compared to cells treated with ATRA, EC19 and EC23, was performed. The most significant increase in Cytochrome-C protein expression level was demonstrated in EC19-treated cells followed by EC23 treatment and finally ATRA treatment in comparison to DMSO-treated control cells. The values were considered statistically significant in comparison to untreated control at ** *p* < 0.01, *** *p* < 0.001. Results are expressed as the mean ± SEM (*n* = 3).

It is relevant here to report the morphological changes observed under the light phase inverted microscope after the exposure of Caco-2 cells to retinoids (specifically, EC19). Rounding of cells was observed in all retinoid-treated cultures (black arrows in light field images in Figure 10A with a higher extent in EC19, in addition to the cytoplasm condensation and the characteristic membrane “blebbing” [49,50]. Moreover, loss of contact, shrinkage, and formation of apoptotic bodies were observed as indications of morphological markers of apoptosis (Figure 10A) [51,52]. 

Cyt-C release is believed to occur following selective permeabilization of the mitochondrial outer membrane and therefore, it is considered a point of no return during cell death [53]. Thus, immunocytochemistry for cytosolic Cyt-C is a pivotal tool for understanding and characterizing the mitochondrial apoptosis pathway and the overall associated morphological changes. We have investigated the retinoid-induced release of Cyt-C from mitochondria to the cytosol by labeling the Cyt-C protein with a cytosolic green-fluorescent antibody and the cells’ nuclei with Hoechst counter stain. The results show that the amount of Cyt-C protein in cytosol of retinoid-treated Caco-2 cells increased after 24 h (Figure 10A) compared to control cells treated with 0.1% DMSO. For quantitative analysis, the current techniques available to the assay of the Cyt-C release rely on cellular extraction followed by Western blotting, immunocytochemistry, or the subcellular localization of GFP-tagged Cyt-C [54]. Western blotting was used in this study; however, it is often difficult to quantify proteins on X-ray film if most of the apoptotic cells have cytoplasmic Cyt-C. Therefore, immunocytochemistry was used to assess the quantitative expression of the cytosolic fraction of Cyt-C using the high technology confocal microscopy and high sensitivity of Alexa Fluor 488 for quantitative analysis [55]. EC19 showed a significant increase in the Cyt-C protein released and number of positive cells expressing the marker compared to control cells (Figure 10B). This quantitative assessment represents the mean anti-Cyt-C antibody green fluorescence intensity emitted from all the cells scanned in the field. EC23 came in the second rank for the induction of Cyt-C protein expression and the intensity followed by ATRA (Figure 10B).

## 3. Discussion

### 3.1. Potent Antiproliferation with Minimal Cytotoxicity of EC-Synthetic Retinoids

ATRA is shown to induce differentiation and inhibits the growth of many types of cancer [15]. Regulatory and cytotoxic effects are mediated through several nuclear receptors and downstream mediators. However, cancer cells can rapidly develop resistance to natural retinoids [21]. Additionally, a larger dose from ATRA above the pharmacological dose (10 µM) is required to reach 50% growth inhibition for different cancer cell lines and this was matched with our data results across several cancer cell lines. These observations prompted the demonstration of previously documented two synthetic derivatives of ATRA; EC19 and EC23 for the induction of neuro-differentiation, to inhibit the proliferation of many cancer cells, with shedding the light on their potency and cytotoxic adverse effects [27]. Several cancer cell lines were used of different origins including hepatocellular, breast, lung, prostate, and lung cancer cell lines in order to identify the most sensitive cancer type to EC19 and EC23. Cell viability assay identified EC19 as the most potent and selective to three cancer cell lines (Caco-2, HepG2 and MCF-7) whereas EC23 was selective to two cell lines (HepG2 and MCF-7). The selectivity index (SI) as marker of cytotoxic adverse effect was calculated in comparison to non-cancerous WI-38 cells, and it was found that both EC19 and EC23, but not ATRA, can be used in lower doses to preferably target cancer types such as Caco-2, HepG2 and MCF-7 cells. 

### 3.2. Caco-2 Cells is In Vitro Model for Further Anticancer Screening

Caco-2 cells was chosen for further biochemical investigations in our study due to many reasons; (1) the global need for novel drugs in this type of cancer. (2) The number of FDA-approved drugs for the treatment of breast cancer and hepatocellular carcinoma greatly outweighs those approved for colon and CRC management and, hence, there is obvious scarcity in CRC cures. (3) Many of the drugs approved for the treatment of CRC are monoclonal antibodies with relatively high cost compared to repurposing available molecules in market such as EC-synthetic retinoids. (4) The major obstacle in the current available anticancer agents is their cytotoxicity to normal cells while treating CRC [15,56]. (5) CRC is one of the most frequently diagnosed cancer types in both men and women and is the third most common cause of cancer mortality worldwide [57]. Finally, specific modulation in signaling pathways in Caco-2 cells including retinoid pathways is associated with metastasis, tumorigenesis as well as resistance to retinoids treatment and progression from normal colon mucosa to carcinoma. Hence, all these reasons were important, interesting and challenging to understand the potency and antiproliferative mechanism of EC19 and EC23 in Caco-2 cells.

### 3.3. A Promising Synergistic Combination with 5-FU in Caco-2 Cells

It was also interesting to consider whether EC-synthetic retinoids would reveal similar synergistic, antagonistic, or additive effects when combined with 5-FU chemotherapeutic agent. Despite the promising results in the treatment of many cancers, the doses required for successful therapy with ATRA are often toxic, leading to “hypervitaminosis-A syndrome” [58]. A possible solution to this problem is to combine ATRA, or other structurally related retinoids, with other chemotherapeutic agents. Co-administered drugs may either reduce or enhance the efficacy of each other’s depending on the therapeutic window of the combined drugs [59]. Although the antimetabolite 5-fluorouracil (5-FU) has been the only drug available to successfully improve 12-month survival in CRC patients, clinical resistance is continuing to be a challenge to date [60]. The combination strategy has become especially important in CRC due to frequent resistance to 5-FU and different anti-cancer drugs that are administered at or close to the maximally tolerated dose [61,62]. Studies showed a synergistic cytotoxic effect of ATRA when combined with 5-FU in CRC and other cancer types [63,64]. Isobologram analysis was an important indicator to provide us with the evidence for drug interactions [65]. Results showed promising synergistic effect of the retinoids in combination with 5-FU on the proliferation of Caco-2 cells with a reduction in all combined IC_50_ values by about 11–17%. Also, DRI and CI confirmed this synergistic effect. Our suggested hypothesis is that synthetic retinoids can act in a way similar to ATRA and can work together with 5-FU by different molecular pathways mainly through the action on RARs nuclear receptors to achieve their overall cytotoxicity to cancer cells. RARs were shown to be members of nuclear receptors that modulate different pathways controlling cancer growth and metastasis [66] like apoptosis [67], kinases-dependent pathways [68] and others. Unlike many chemotherapeutic drugs, synthetic retinoids such as EC19 and EC23 can be active at lower doses and over longer periods of incubation due to the environmental and cellular stability [27,69] which could further support their additive effects. Hence, EC-synthetic combinations would be more beneficial than ATRA in clinical settings due to their observed potent effect in relatively lower doses than individual standard retinoids. This may pave the way for future in vitro and in vivo combination strategies that impact patient overall survival, reduce the possible side effects and optimize the treatment outcomes.

### 3.4. The Antiproliferative Effect of EC19 and EC23 in Caco-2 Cells is Due to Early Apoptosis and Cell Cycle Arrest

Determining the mechanism of actions of EC- synthetic retinoids in caco-2 cells was essential for advancing our understanding of these compounds in the sake of effective therapeutic alternatives. Many studies suggested that the effects of retinoids on cell proliferation, cell cycle, and apoptosis are either retinoid specific or cell-type specific [70,71,72,73]. These findings led to the successful clinical application of different retinoids in the treatment or prevention of various human cancers including Caco-2 cells. Also, studies in Caco-2 cells and other cell types suggest that ATRA and other natural isoforms cause arrest of cells in the G_1_/S phase of the cell cycle and increase the percentage of cells in G_0_-G_1_ boundary [70,74,75]. This observation has been demonstrated in our results as ATRA and EC19 were able, after 12-h incubation, to induce slight cell cycle arrest in both S- and G_2_/M phases (this effect was exaggerated after 24 h), in addition to subG_0_-G_1_ phase arrest. This effect may arise from the integration between the retinoid-induced differentiation and apoptosis since retinoic acid-induced phenotypic differentiation at early-stage and late apoptosis is time- and concentration-dependent [76,77]. In terms of the potency, EC19 and EC23 were able to significantly reduce the percentage of viable cells and increase the early apoptotic cells compared to both ATRA and negative control. This was confirmed by the calculation of AI for both EC19 and EC23. These results are matched with the difference observed in antiproliferative potency of EC19 and EC23 on Caco-2 cells and may suggest their apoptotic mechanistic effect. The apoptotic effect was previously documented for ATRA and many synthetic retinoids [71,73].

### 3.5. Differential Transactivation of RARs Explains the Differential Sensitivity of Caco-2 Cells

In a previous report, ATRA was shown to have a pro-differentiating effect in Caco-2 cells [78], probably by stimulating the expression of RAR-β via a retinoic acid response element (RARE) [79]. Furthermore, the expression of RAR-β is downregulated in many cancers, including colon cancer [80]. The findings of the present study fit into the context of these results. We found that ATRA selectively enhances the mRNA level of RAR-β after 24 h. Similarly, EC19 stimulates the expression of RARβ after 24 h; however, the effect at IC_50_ appears to be much higher than ATRA (approximately 10-folds higher) in contrast to EC23 that did not show any significant increase in RAR-β. Transactivation of RAR-β gene has been previously linked to the cellular arrest and apoptosis in subG_0_-G_1_ phase in a wide variety of cancer types including CRC [81]. Hence, natural retinoids have RAR-β-based tumor-suppressive activity, and loss of normal RAR-β function is associated with the progression of a diverse range of cancers [82]. For instance, RAR-β activity was markedly increased in HT-29 cells exposed to ATRA leading to the activation of p53/p21 signaling pathway [83]. The activated p21^Waf1/Cip1^ inhibits the cyclin-dependent kinases (CDKs) and consequently induces cellular subG_0_-G_1_ arrest. These observations may suggest that the reduction in cellular proliferation observed during the viability and apoptosis assays was due to subG_0_-G_1_ arrest downstream of RAR-β stimulation. Moreover, RAR-α is expressed mainly in adult tissues while RAR-γ is expressed in skin tissues [84] and their gene overexpression in tumor cells leads to growth inhibition and accumulation of cells in the subG_0_-G_1_ phase of the cell cycle [84,85,86]. These differential activities may explain the enhanced antiproliferative activity of EC19 due to both RAR-β- and RAR-γ- mediated activity compared to only RAR-γ- dependent action by EC23. There are comparable cellular mediated results of ATRA and EC19 on both RAR-β- and RAR-γ to our previously reported molecular selectivity of these molecules to these receptors while EC23 was different in response which may be due to other resistance factors in Caco-2 cells to activity of EC23 molecule [28].

### 3.6. Phenotypic Perturbations Seen under Microscope are Pertinent to Apoptosis Induction

The morphological observation confirmed the apoptotic effect where control cells treated with media containing 0.1% DMSO were packed as a tight and adherent monolayer to the well surface. On the other side, Caco-2 cells exposed to retinoids, particularly EC19, showed obvious cytoplasmic condensation, characteristic membrane “blebbing”, loss of contact, shrinkage, and formation of apoptotic bodies. These changes are indicative of apoptosis with different extents according to the potency of each compound [49,50].

### 3.7. The Molecular Changes of EC-Synthetic Retinoids on Transcriptomic and Proteomic Levels

Tracking and understanding the mechanism of apoptotic activity of EC-synthetic analogues on the genetic level is important to conclude their potential use in further in vivo studies and future clinical applications. Apoptosis is a major anticancer mechanism that is initiated through one of two main pathways: the intrinsic and the extrinsic pathways. The intrinsic pathway is called mitochondrial apoptosis where the cell kills itself through the activation of intracellular mediators that loosen the mitochondrial membrane and induce the release of mitochondrial contents [87]. The other form is extrinsic apoptosis where cells sense the stress caused by signals from the external environment [88]. Caspases play an important role in anticancer drug mechanisms to create an overlapping hub between the two signaling pathways in response to a specific drug to eliminate cancer cells. In this regard, the ability of EC19 to provoke apoptosis in Caco-2 cells resulted in a significant up-regulation of the expression level of caspase-8, the leading early caspase type in the extrinsic pathway which converges to caspase-3 as a key executioner of apoptosis [89]. The protein expression level of caspase-3 was significantly increased with EC19, matching what was seen in the gene expression assay. Moreover, Bcl-2 and Bax are two discrete members of the family of genes involved in resistance to many apoptotic stimuli including the most conventional cytotoxic anticancer drugs [90,91]. Bax and Bcl-2 genes are responsible for switching on/off apoptotic mechanisms and are considered as important gatekeepers to the apoptotic response. While Bcl-2 gene is functionally characterized as an apoptosis-suppressing factor, the Bax protein is more functionally characterized as an apoptosis-promoting factor. Therefore, the intracellular Bax/Bcl-2 ratio can profoundly influence the ability of a cell to respond to an apoptotic signal [92,93]. EC19 was able to induce up-regulation of the expression level of the pro-apoptotic Bax gene with about 2 fold higher than control and this was confirmed on protein expression level as well.

### 3.8. The Modulation of Cyt-C Expression with EC-Synthetic Retinoids Treatment is Indicative of Intrinsic Apoptosis Induction

The activated Bax binds to the mitochondrial outer membrane leading to the opening of the mitochondrial voltage-dependent anion channel (VDAC) followed by the release of Cyt-C from the mitochondria into the cytosol where it activates the caspase-dependent signaling and subsequent apoptosis. Therefore, the Cyt-C release from mitochondria is an indicator of activation of the intrinsic apoptotic pathway [94]. Herein, we assessed the expression level of Cyt-C on the gene and protein levels in order to assure the adoption of the intrinsic pathway for apoptosis induction [95]. The level of the Cyt-C transcription was induced quickly after 24 h with a higher level for ATRA followed by EC19 and EC23. However, Cyt-C protein level detected using Western blotting and immunocytochemistry showed a significant increase with EC19 compared to other retinoids. This difference in observation despite the optimization of procedures might suggest some explanations; first, for the intrinsic pathway of apoptosis induced by EC19, the gene expression of Cyt-C is not the major determinant of its protein level, and there might be other contributing factors controlling the potency of EC19 in apoptosis. Also, EC19 might have an effect on other regulatory processes controlling mRNA translation, miRNA targeting species and post-translational modifications, like phosphorylation, acetylation and glycosylation and hence, increasing the stability of mRNA and protein products and this is something we are working-on currently in the project [96].

### 3.9. EC19-Induced Apoptosis in Caco-2 Cells Involves the Induction of p53 Protein Expression 

Another major apoptosis signaling pathway involves the p53 tumor suppressor protein [97]. The ability of p53 to control apoptosis in response to abnormal proliferative signals and stress is crucial for its tumor suppression role. The p53 is a nuclear transcription factor that regulates the expression of a wide variety of genes involved in apoptosis. It is able to induce Bax oligomerization and Cyt-C release from mitochondria [98]. Gene expression analysis of p53 did not show any significant increase in its level after 24 h; however, the protein expression level was significantly increased with EC19 and this might suggest the preferential role of EC19 on the stability of protein product compared to other retinoids. Overall, the EC19 predominant apoptotic effect is due to stable over-expression of intrinsic key genes and proteins of apoptosis including Bax, p53, Caspas-3, Cyt-C.

### 3.10. The Reduction of the Intracellular Glutamate Pool May Further Explain the Superior Activity of EC19 over EC23 in Caco-2 Cells

Glutamate is an essential molecule for cells, especially cancer cells due to its paracrine effect on intracellular and extracellular communications where even the small changes in its levels significantly impact the cellular transformation and cancer progression [99,100,101]. The extracellular glutamate is mainly required for intercellular communication and activation of survival pathways, like the MAPK and PI3K pathways, promoting growth and invasion [101,102]. Also, glutamate is the most abundant amino acid used by highly proliferating cancer cells to increase proliferation since it is converted to α-ketoglutarate (α-KG) and channeled into the TCA cycle [103]. Moreover, glutamate has an important role in the creation of cellular building blocks for cancer cell growth such as nonessential amino acids and nucleotides to survive under metabolic stress conditions [104,105,106]. Finally, glutamate is required for intracellular glutathione production, which is a pivotal molecule for an adequate oxidative status of cancer cells [107]. Therefore, depleting the cellular stores of glutamate ameliorates cellular defense against reactive oxygen species (ROS), reduces intercellular communication through glutamate-dependent signaling pathways and may lead to lower levels of ATP and essential nutrients [108]. Previous studies showed an interesting negative correlation between glutamate enrichment and activation of retinoid signally pathway of RARs, RXRs, and peroxisome-proliferator-activated receptor (PPAR) pathways [109]. These nuclear receptors act as partners for dimerization and translocation to the nucleus for deactivating the glutamate enrichment and tumorigenesis [109]. Any deregulation in the function of these nuclear receptors, especially RAR-α and -β, can be associated with glutamate formation and release [109]. Hence, the concentration of extracellular and intracellular glutamate plays an important role to understand if the antiproliferative activities of retinoids are mediated, at least in part, through the inhibition of glutamate synthesis and secretion [105,110]. Therefore, a glutamate assay is an important tool that further confirmed EC19 potency as an antiproliferative agent since it was able to reduce 40% of the total intracellular glutamate and consequently lower the amount of glutamate that was released extracellularly (unlike ATRA and EC23). Moreover, because the glutamine/glutamate ratio is controlled by glutaminase and glutamine synthetase [107], the interference with glutaminase activity could be a possible explanation for the reduction of glutamate levels. Nevertheless, further investigation is required to accurately confirm the main players controlling the cellular glutamate metabolism.

### 3.11. EC-Synthetic Retinoids are Pro-Oxidants that Promote Cellular Vulnerability to Oxidative Stress

Oxidative stress may be an early event in the activation of the apoptotic machinery [111]. Proteins and DNA are prone to mutations and/or damage by oxidative stress causing different pathological conditions. This is evident when ROS increase in cancer cells, such as CRC, where they were found to be cytotoxic. Thus, ROS-mediated DNA damage halts the malignant transformation of cancer cells and suppresses tumorigenesis [112]. Therapeutic candidates that promote cellular pro-oxidant state could favorably destroy CRC cells through enhancing cellular oxidative stress. Here, both EC19 and EC23 significantly reduced the cellular TAC. The ability of these compounds to significantly reduce cellular TAC may be connected with their anti-tumorigenic outcome as the cells become more prone to pro-oxidants. Additionally, the glutamate depletion, which is a major determinant for the concentration of one of the most pivotal antioxidant molecules (i.e., GSH), may explain, at least in part, the profound potency of EC19 for the reduction in total antioxidant capacity in Caco-2 cells [113,114,115].

### 3.12. Retinoids Reduce the Metastatic Potential of Caco-2 Cells via Modifying the Expression of Relevant Genes

The interaction between primary tumor cells and host vasculature is an early step during tumor development and results in poor prognosis owing to the active migration of tumor cells into adjacent tissues through metastasis and invasion [116]. During the process of invasion, tumor cells penetrate basement membranes either to reach the interstitial stroma or to gain access to bloodstream [117]. Retinoic acid has been shown to reduce the cell-cell and cell-matrix adhesion of human intestinal epithelial cells to culture flasks and plastic dishes [117]. Also, retinoids were shown to reduce the metastatic potential of ATRA-resistant colon cancer cells via a RAR-independent mechanism that involves reduced matrix metalloproteinase (MMP) mRNA levels and activity [118]. Various degradative enzymes, either in the tumor cells or in host cells, are induced to do so by tumor-derived factors related to retinoid signaling such as *RAI2* or through retinoid-independent metastasis modulators such as *WRN*, IGF1-R, TGF-β1 and E-cadherin.

IGF1-R is often elevated in many cancer tissues including colon cancer and its activation has been linked to increased NF-κB activation and thus proliferation, survival, metastatic potential, and angiogenesis [119,120]. Therefore, targeting IGF-1R is a promising approach in anticancer therapeutics. Transforming growth factor-beta (TGF-β) is another potential contributor to both angiogenesis and CRC tumor progression [121]. TGF-β has a characteristic mechanism of activation through binding a dimer of type II receptors (TGFβR-II) leading to the recruitment of TGFβR-I (ALK5) and consequent phosphorylation of R-Smad2 and R-Smad3 [122]. These R-Smads complex with Smad4 and associate with transcriptional factors in order to regulate the expression of target oncogenes controlling Caco-2 cell growth, invasion, epithelial to mesenchymal transition, evasion of immune surveillance, and metastasis [123,124].

E-cadherin is another invasion-orchestrating marker that acts as a core component of epithelial adherent junctions and, thereby, essential for colon tissue development, differentiation, and maintenance [125]. The colon is lined by an epithelial monolayer secreting E-cadherin that acts as a signaling epicenter regulating cell behavior in response to intra- and extra-cellular stimuli. In colon cancer, E-cadherin has been extensively studied and linked with many pathways including Wnt/β-catenin signaling; therefore, dysregulation of this pathway is a predominant driver of colon tumorigenesis [126]. Two contradicting roles have been reported for the E-cadherin; the first is a predominant tumor-suppressing role to interfere with the pro-tumorigenic transformation that is promoted by β-catenin-activating mutations as well as a role in intracellular signaling that ultimately inhibits metastasis [125].

Retinoic acid-induced 2 (*RAI2*) has been recently identified as a transcriptional regulator that controls the expression of several key regulators in cancer [127]. Low expression of *RAI2* was detected in breast and CRC cancers and it was associated with early-onset bone metastasis in ERα-positive breast malignancies [127,128]. In colon cancer, restoration of *RAI2* expression suppressed CRC cell proliferation, migration, and invasion, as well as induced programmed cell death [40]. Also, the Werner *(WRN)* gene, which is a human RecQ DNA helicase with both a 3′ to 5′ helicase activity and a 3′ to 5′ exonuclease activity [129], has been identified as a tumor suppressor gene where any germline mutation in its sequence can cause Werner’s syndrome (WS) with an increased risk of tumor development [130]. A recent study highlighted its role in CRC and stated that the downregulation of *WRN* is “highly associated” with late-stage CRC. A possible link between the *WRN* gene and the intracellular cell signaling of retinoids has been postulated to be a key regulator of retinoic acid-induced stem cell differentiation [131]. Moreover, *WRN* is among the key regulators of p-53-mediated transcriptional activity. High *WRN* expression induces the transcription of p53-orchestrated genes leading to enhancement of p53-mediated cell cycle arrest and subsequent apoptosis [132].

Our results were in agreement with the potential roles of these aforementioned networking mediators where the exposure of Caco-2 cells to ATRA and EC-synthetic analogues restored the expression of E-cadherin, *RAI2* and *WRN* genes with significant downregulation of IGF1-R and TGF-β. EC19 had the most potent activity in inducing the over-expression of E-cadherin and *WRN* genes whereas ATRA was mainly a potent inducer of *RAI2*. Moreover, the invasion assay confirmed the results as all retinoids were able to inhibit the invasion of Caco-2 cells through the collagen matrix membrane and EC19 showed the highest inhibitory activity in a time-dependent manner followed by EC23 and ATRA.

## 4. Materials and Methods

### 4.1. Retinoids Solution

EC19, EC23, and ATRA were purchased as ready to use powder from (Clinilab, Tocris Biosciences, UK; purity ≥ 98% (high-performance liquid chromatography, HPLC)) and stock solutions were prepared in DMSO (Sigma-Aldrich) as a concentration of 1mM. Aliquots from the stock solution were stored at −20 °C freezer. ATRA stock solution and different working concentrations aliquots were kept away from light during storage and along the experiments. 

### 4.2. Cell Culture

All cancer and normal cell lines used in this study were purchased from the cell culture bank in tissue culture unit at the Holding company for the production of vaccines, sera, and drugs (VACSERA, Giza, Egypt). Different types of cancer cell lines were selected including hepatocellular carcinoma (HepG2 and Huh-7), breast cancer (MCF-7 and MDA-MB-231), colorectal carcinoma (Caco-2, HCT-116 and HT-29), lung cancer (A549), and prostate cancer (Du-145 and PC3), in addition to two normal cell lines: healthy African green monkey kidney cells (Vero) and normal human lung fibroblasts (WI-38). All cell lines were maintained in the Center of Scientific Excellence “Helwan Structural Biology Research, (HSBR)” under standard laboratory conditions. Standard culturing conditions were performed by using culture media supplemented with 10% fetal bovine serum (FBS) and 1% penicillin/streptomycin, and the cells were incubated at 37 °C in a humidified atmosphere containing 5% CO_2_. Additionally, all cell cultures were handled carefully in reduced light conditions to account for the photosensitivity, and thus instability, of ATRA. 

### 4.3. Antiproliferation assay and Selectivity Index (SI) Calculation

The antiproliferation and cytotoxicity were assessed using 3-(4,5-dimethylthiazol-2-yl)-2,5-diphenyltetrazolium bromide (MTT, Serva) colorimetric assay [133]. To investigate the antiproliferation, all cell lines were seeded at a density of 20,000 cells per well in 96-well plates and allowed to adhere overnight. The attached cells were treated with five series concentrations (0.01, 0.1, 1, 10, and 100 μM) of synthetic retinoids (EC19 and EC23) and ATRA (standard retinoid positive control) in triplicate. These concentrations were prepared by diluting the stock solutions with serum-free culture media. The negative control cells were those treated with 0.1% DMSO solvent alone. The treated cells were handled in reduced light and maintained under standard culture growth conditions for 24 h. After the incubation period, MTT powder was prepared as a stock solution (5 mg/mL) and 20 μL of the MTT working reagent (final concentration 0.5 mg/mL) was added to each well. This was followed by incubation with MTT reagent for 4 h in a humidified atmosphere (37 °C, 5% CO_2_) with subsequent addition of 150 μL of DMSO solubilizing solvent and incubation for 20 min. The absorbance of solubilized violet formazan crystals was measured at 540 nm with Biotek 800 TS microplate reader. IC_50_ was calculated as the concentration of retinoid that produced 50% cell growth inhibition. For cytotoxicity assessment, WI-38 normal human fibroblast cells were used for the calculation of SI which is the ratio of IC_50_ retinoid (WI-38)/IC_50_ retinoid (cancer cell line) [134]. Increasing SI value above 1 indicates a more effective and safer drug as an anticancer compared to normal tissues [135].

### 4.4. Isobologram Analysis of Combined Drug Effect

5-fluorouracil (5-FU) is a standard chemotherapy with known activity on Caco-2 cells and reported side effects [136,137]. The previously mentioned procedure of antiproliferation assay was used to evaluate the combined effects of retinoids and 5-FU to enhance the chemo-sensitivity of drugs and reduce the toxic IC_50_ doses. Cells were treated with a fixed ratio of combined agents simultaneously at doses that typically corresponded to 4, 2, 1, 0.5, 0.25 and 0.125× times the individual IC_50_ values of each retinoid and 5-FU. The results were quantified using “multiple-drug effect analysis” based on the isobologram technique originally developed by Chou and Talalay [138,139]. This mathematical model allows the determination of CI (combination index) values based on the Equation (1) [140]:

CI = d1/D1 + d2/D2 (1)

where d1 and d2 are the doses of drug 1 and drug 2 that, when given in combination, produce a specific response. D1 and D2 are the doses of drug 1 and drug 2, that, when given individually, produce the same response. Also, CI < 1 is synergy, CI = 1 is additivity and CI > 1 is antagonism

The DRI is defined as “the fold decrease in the dose of each drug if two drugs are given in combination, as opposed to individual treatment, to achieve a particular level of cytotoxicity” and is calculated from the Equation (2) [141]:

DRI (for drug 1) = D1/d1 (2)

DRI > 1 reflects favorable dose reduction and consequently greater DRI value means a more favorable drug combination. DRI < 1 indicates unfavorable dose reduction and ineffective combination. DRI that equals to 1 means there was no reduction in the dose for the selected drug [142].

### 4.5. Detection of Apoptosis Using Annexin V (AV)/Propidium Iodide (PI) Assay

The ability of synthetic retinoids to induce apoptosis in one of the sensitive cancer cell lines (namely, Caco-2 cells) was detected by Annexin V-FITC and PI according to the protocol of the detection kit (Beckman Coulter, Brea, CA, USA) [143]. In brief, cell suspension was cultured in 25 cm^2^ flasks overnight and then a number of flasks was divided into groups and treated with the IC_50_ concentration of retinoids for either 12 or 24 h. Cells were collected, washed with phosphate-buffered saline (PBS) (Lonza), and resuspended in 1 mL of binding buffer. 100 µL cell suspension was incubated with 1 µL FITC-labeled Annexin V and 5 µL PI for 15 min at 4 °C in the dark. Finally, 400 µL ice-cold binding buffer was added, and the apoptotic cells in each sample were analyzed by Beckman Coulter Epics XL flow cytometer. Apoptotic index (AI) was calculated as the number of apoptotic cell deaths expressed as a percentage from the total gated cells [144,145].

### 4.6. Cell Cycle Analysis

The cell cycle analysis was performed using flow cytometry (Beckman Coulter, Brea, CA, USA). Briefly, 1 × 10^6^ Caco-2 cells were seeded into a number of 25 cm^2^ flasks overnight which were then divided into two groups and treated with IC_50_ of retinoids for either 12 or 24 h. After incubation, cells were harvested, fixed with 70% alcohol overnight, and stained with PI (50 µg/mL). DNA content was measured by Beckman Coulter Epics XL flow cytometer and G_0_/G_1_, S, and G_2_/M cells were gated as appropriate using Flowing Software 2.5.1 (Turko Bioimaging, Turku, Finland). 

### 4.7. Gene Expression Analysis of Multiple Signaling Key Genes

Sensitive cancer cell line (Caco-2) was used for gene expression analysis of different genes controlling differentiation, apoptosis, tumor resistance, aggressiveness, and metastasis. Cells were seeded overnight at a density of 1 × 10^6^ cells per well in 6-well plates in triplets at the standard incubation conditions, and then treated with IC_50_ concentrations of retinoids for 24 h in addition to the DMSO-treated cells (i.e., solvent control). After incubation, cells were harvested, and the total RNA was purified using Favor-Prep^TM^ Blood/Cultured cell total RNA purification mini kit (Favorgen Biotech Corp., Ping-Tung, Taiwan). RNA was reverse transcribed into the first-strand cDNA using Revert Aid First Strand cDNA Synthesis Kit (Thermo Scientific, Waltham, MA, USA). HERA^PLUS^ SYBR^®^ Green qPCR Kit (Willowfort, Nottingham, UK) was utilized in quantitative polymerase chain reactions (qPCR). Gene expression was analyzed and quantified using the 2ˆ^−ΔΔCT^ method [146]. The sequences of primers used are listed in Table S1.

### 4.8. Western Blotting

The expression of Bax, Bcl-2, Cyt-C, cleaved cas-3 and p53 proteins in Caco-2 cells was determined using Western blot procedure according to the methods mentioned previously [147,148]. In brief, 1 × 10^6^ cells per well in 6-well plates in triplets were treated with the IC_50_ dose of ATRA, EC19 or EC23 for 24 h. Then, cells were lysed in 150 µL pre-cold lysis buffer [Tris-Base “10 mM”, NaCl “100 mM”, Ethylene glycol bis (2-aminoethyl) tetraacetic acid (EGTA) “20 mM”, ethylenediaminetetraacetic acid (EDTA) “25 mM”, 1% (*v*/*v*) NP-40, and 1% (*v*/*v*) Triton X-100; pH 7.4] immediately supplemented with 1:450 protease and phosphatase inhibitors cocktail (Sigma-Aldrich). Caco-2 cells were immediately collected by cell scraper, sonicated, and centrifuged at 12,000 rpm for 15 min. Total proteins in the produced supernatant were determined colorimetrically using the Pierce™ 660 nm Assay (Thermo Scientific, Rockford, IL, USA). Equal amounts (30 µg) of protein were mixed with sodium dodecyl sulfate (SDS)-loading buffer containing Tris-HCl, dithiothreitol (DTT), SDS, glycerol, and Bromophenol blue. The protein samples were then denatured by boiling for 5 min, allowed to cool on ice for 10 min and separated by SDS-polyacrylamide gel electrophoresis (Cleaver Scientific Ltd., Rugby, Warwickshire, UK), and transferred onto Polyvinylidene Difluoride (PVDF) membranes for 30 min using Trans-Blot^®^ SD semi-dry transfer cell (Biorad). Membranes were then blocked with 5% (*w*/*v*) blotting-grade dry milk (Biorad) in Tris-buffered saline-Tween-20 (TBS-T), washed and incubated with antibodies against Bax, Bcl-2, cleaved caspase-3 (1:100–1:2000, Cell Signaling Technology), cytochrome c (1:500, Santa Cruz Biotechnology), p53 (1:1500, Abcam) and β-actin (1:2500, Sigma-Aldrich) for 16–17 h at 4 °C in a humidified chamber. The blots were washed, incubated at RT with matched horseradish peroxidase (HRP)-linked secondary antibodies (Dako, Denmark) for 1.5 h, and the bands were visualized with chemiluminescence Western Lightning ECL (Perkin Elmer, Waltham, MA) for 1 min in Chemi-Doc imager (Biorad, Hercules, CA, USA) and finally analyzed with Bio-Rad Image Lab software with normalization to β-actin.

### 4.9. Cell Invasion Assay

Caco-2 cell invasion was assessed using a QCM™ 24-well Colorimetric Cell Invasion Assay kit (Chemicon International, Temecula, CA, USA (Cat # ECM551)), according to the manufacturer’s instructions. Cells were starved of serum for 24 h. Then, cells were collected, suspended in serum-free Dulbecco’s Modified Eagle Medium (DMEM) growth media containing either IC_50_ dose of retinoids or 0.1% DMSO (negative control), and seeded into the upper chamber of inserts at a density of 2.5 × 10^5^ cells per insert and divided into two groups for either 24 or 48 h. Inserts were further added into a 24-well plate containing 500 µL of 10% FBS-containing DMEM per well. After incubation for 24 or 48 h at 37 °C, remaining media was removed from the invasion chamber, and cells that had migrated to the other side of the membrane were stained with “Cell Stain” solution for 20 min at room temperature. Non-invaded cells were carefully removed from the upper side of the chamber with the help of cotton-tipped swabs. The stain of invaded cells was then extracted by 200 µL of extraction buffer in a clean well for 15 min at room temperature. Finally, 100 µL extracted stained solution was transferred into a 96-well plate and the optical density was measured at 490 nm with BioTek 800 TS microplate reader.

### 4.10. Measurement of Intracellular and Secreted Glutamate

The glutamate assay was performed using the Glutamate Assay Kit (Sigma-Aldrich, MAK004, St. Louis, MO, USA) according to the manufacturer’s protocol [149]. In brief, 1 × 10^6^ Caco-2 cells were seeded in 6-well plates in triplicates with 1-mL culture medium overnight and then treated with IC_50_ of retinoids in glutamate-free culture medium for 24 h. Cells were lysed using 100 µL glutamate assay buffer and insoluble cell debris was then removed by centrifugation at 13,000× *g* for 10 min. The supernatant was transferred to a 10-KDa molecular weight cut-off (MWCO) spin filter (Sigma-Aldrich, St. Louis, MO, USA) to remove any particulates in the sample, and then was brought to a final volume of 50 µL with glutamate assay buffer and transferred to a 96-well plate. Finally, 100 µL of reaction mix was added to each well and the mixture was incubated for 30 min at 37 °C. The optical density was measured at 450 nm using BioTek 800 TS microplate reader (BioTek, Winooski, VT, USA). For the measurement of secreted glutamate, 50 μL samples of extracellular culture medium were placed in a 96-well plate and the kit’s enzymatic reaction mix was added to induce a glutamate-dependent color change. After 30 min incubation, the reaction products were read at the same wavelength. For determination of glutamate concentration (nmole/µL), a calibration curve was constructed using supplied 0.1 M glutamate standard with different dilutions (0 (blank), 2, 4, 6, 8 and 10 nmole per well). Glutamate concentration was measured according to the manufacturer’s instruction and as documented in earlier studies [150,151,152,153].

### 4.11. Total Antioxidant Capacity (TAC)

Total antioxidant capacity was assayed spectrophotometrically using a Biodiagnostics^®^ kit (Catalogue number TA 25 13, Biodiagnostics, Cairo, Egypt). Briefly, 1 × 10^6^ Caco-2 cells per well were seeded in 6-well plates overnight, and then treated with the IC_50_ of retinoids for 24 h. The measurement of total antioxidants is based on treating the cell lysate with a known excess of H_2_O_2_ (50 µM) [154]. A certain amount of H_2_O_2_, equivalent to TAC, is eliminated leaving a residual amount of H_2_O_2_ that is quantified colorimetrically by the enzymatic conversion of 3, 5, dichloro-2-hydroxy benzene sulphonate to a colored product.

### 4.12. Immunocytochemistry (ICC) and Detection of Morphological Changes

Caco-2 cells were seeded onto coverslips at a density of 1 × 10^6^ cells using 0.1 mg/mL Poly-L-Lysine (Sigma-Aldrich, St. Louis, MO, USA) for immunocytochemistry (ICC) analysis. After treatment with IC_50_ of retinoids, cells were fixed in formalin neutral buffer (FNB) solution. The fixed cells were permeabilized with 0.1% Triton-x 100 and then blocked in 1% bovine serum albumin (BSA, prepared in PBS containing 0.1% Tween 20) for 30 min. Then, cells were incubated with Alexa Fluor^®^ 488-conjugated anti-cytochrome C antibody overnight. After incubation, nuclei of cells were stained with Hoechst 33,258 (Sigma-Aldrich, St. Louis, MO, USA, 1:500). The coverslips were mounted in anti-fading medium (0.1% p-phenylenediamine, dissolved in 90% glycerin/PBS) and cytochrome C protein expression was detected using Carl Zeiss LSM 710 confocal microscope. In addition, Caco-2 cell morphology was observed after treatment with IC_50_ for 24 h and representative images were captured by an inverted phase-contrast microscope (OPTECH Biostar IB) to detect the morphological changes.

### 4.13. Statistical Analysis

Statistical analysis was performed using GraphPad Prism 7 analysis software (GraphPad, San Diego, CA, USA). The significance of the data was analyzed using one-way analysis of variance (ANOVA). *p* values of *p* < 0.05 (*), *p* < 0.01 (**), and *p* < 0.001 (***) were considered significant.

## 5. Conclusions

EC19 and EC23 are two novel synthetic derivatives of ATRA that inhibit the proliferation of multiple cancer cell lines. In Caco-2 cancer cells, EC19 was potent to induce cell cycle arrest at subG_0_-G_1_, S and G_2_/M phases. EC19 was able to re-express tumor suppressor genes (*RAI2* and *WRN*) and reduce the total cellular antioxidant capacity with more profound amplitudes than the parent ATRA compound. EC-synthetic retinoids have strong synergistic cytotoxicity in Caco-2 cells co-treated with 5-FU. However, additional in vitro and in vivo studies are needed to scrutinize the downstream pathways by which these retinoids, alone or in combination with standard chemotherapeutics, elicit their actions. Nonetheless, the present study suggests that EC-synthetic retinoids, and particularly EC19, may potentially be effective anticancer candidates for colorectal cancer. However, further in vivo investigations are required to confirm the anticancer activity and to discriminate between the two retinoid analogues in terms of biological potency.

## Figures and Tables

**Table 1:** The In Vitro antiproliferative activity of all-*trans*-retinoic acid (ATRA), EC19 and EC23 against different cancerous cell lines. WI-38 was selected as a normal cell line from human origin for the calculation of SI (selectivity index). SI was calculated as IC_50_ retinoid (WI-38)/IC_50_ retinoid (cancer cell line). Data represent mean ± standard error of the mean (SEM), *n* = 3.

Cell Line	* IC_50_ (µM) ± SEM and Corresponding SI					
	ATRA		EC19		EC23	
	IC_50_	SI	IC_50_	SI	IC_50_	SI
WI-38	34.0 ± 1.1	1	48.4 ± 1.2	1	8.01 ± 0.13	1
Vero	>100	1	>100	1	35.23 ± 1.02	1
HepG2	36.2 ± 1.9	0.94 ± 0.02	42.2 ± 0.92	1.2 ± 0.002	0.74 ± 0.001	10.82 ± 1.0
Huh7	>100	<1	>100	0.14 ±0.01	>100	0.02 ± 0.001
Caco-2	58.0 ± 1.0	0.59 ± 0.01	10.8 ± 0.1	4.5 ± 0.01	14.7 ± 0.73	0.54 ±0.01
HCT-116	>100	<1	>100	<1	44.46 ± 1.1	0.18 ± 0.003
HT-29	0.002 ± 0.01	>1	>100	<1	>100	<1
MCF-7	99 ± 0.26	0.34 ± 0.01	9.4 ± 0.13	5.18 ± 0.1	5.56 ± 0.01	1.44 ± 0.004
MDA-MB 231	>100	<1	>100	<1	>100	<1
DU-145	>100	<1	86.9 ± 2.0	0.56 ± 0.001	>100	<1
PC3	>100	<1	>100	<1	>100	<1
A549	84.7 ± 3.2	0.4 ± 0.01	>100	<1	>100	<1

* IC_50_ values were determined by non-linear regression of dose-response using the 4-parameter logistic model (4PL) in GraphPad prism 7.

**Table 2:** Isobologram analysis of the combinations of individual retinoids and 5-fluorouracil (5-FU) (using fixed ratios of the individual IC_50_ values) in Caco-2 cells. A synergistic effect was observed with the combination strategy. Dose reduction index (DRI) and combination index (CI) confirm the synergistic effect. Data represent mean ± SEM, *n* = 3.

Drug	IC_50_ (µM)	×IC_50_ (µM) (Combination) with 5-FU	Individual IC_50_ (µM) (Combination)		DRI	CI
			5-FU	Retinoid		
5-FU	18.4 ± 0.6	----------------------------------------------------------				
ATRA	58 ± 1.0	0.15 ± 0.01	2.8 ± 0.3	8.7 ± 0.9	6.9 ± 0.7	0.29 ± 0.03 **
EC19	10.8 ± 0.1	0.229 ± 0.03	4.21 ± 0.6	2.47 ± 0.3	4.5 ± 0.6	0.46 ± 0.06 **
EC23	14.7 ± 0.7	0.136 ± 0.02	2.5 ± 0.3	2 ± 0.2	7.5 ± 0.8	0.27 ± 0.03 **

** *p* < 0.01; where *p* values indicate the level of significance compared to CI = 1.0.

**Table 3:** The percentage of viable, apoptotic, late apoptotic, and necrotic cells was measured by AV/PI assay using flow cytometry. The assay was performed after the treatment of Caco-2 cells for 12 or 24 h with ATRA, EC19 and EC23 compared to 0.1% DMSO negative control. The apoptotic index was calculated as the percentage of the apoptotic cells to the total number of gated cells. Data represent mean ± standard error of the mean (SEM), *n* = 3.

Apoptosis Analysis of Caco-2 Cell Line	12 h Treatment				24 h Treatment			
	Control	ATRA	EC19	EC23	Control	ATRA	EC19	EC23
% viable cells (C^−−^)	80.30 ± 7.9	87.99 ± 15.0	93.65 ± 14.0	91.61 ± 13.7	78.83 ± 6.4	52.4 ± 11.5 *	24.5 ± 11.8 ***	34.4 ± 18.3 ***
% early apoptotic cells (C^+−^)	19.61 ± 5.5	11.99 ± 1.9	6.30 ± 1.1	8.34 ± 1.1	20.20 ± 2.8	47.4 ± 2.5 **	75.3 ± 13.8 ***	65.4 ± 18.4 ***
% late apoptotic cells (C^−+^)	0.00	0.00	0.01	0.01	0.0	0.0	0.0	0.0
% necrotic cells (C^++^)	0.08	0.01	0.4 ± 0.1	0.05	0.95 ± 0.18	0.28± 0.17	0.18 ± 0.05	0.16 ± 0.14
% Apoptotic index (AI)	20	12	6.3	8.3	18	63	84	89

* *p* < 0.05, ** *p* < 0.01 and *** *p* < 0.001. *p* Values indicate the significance in comparison to untreated control cells.

**Table 4:** Cell cycle analysis of Caco-2 cells after ATRA, EC19 and EC23 treatment for 12 and 24 h showing the DNA content at different cycle phases. % G_2_M/G_0_-G_1_ ratio was calculated as the percentage of the dividing cells in the mitotic phase to the growing cells in the G_0_-G_1_ phase.

Cell Cycle Analysis of Caco-2 Cell Line	Control	12 h Treatment			24 h Treatment		
		ATRA	EC19	EC23	ATRA	EC19	EC23
% SubG_0_-G_1_	0.24 ± 0.1	0.14 ± 0.1	0.29 ± 0.1	0.46 ± 0.1	1.6 ± 0.19 ***	4.5 ± 0.6 ***	3.9 ± 0.43 ***
% G_0_-G_1_	67.2 ± 10.1	26.1 ± 3.6	39.1 ± 6.7	58.7 ± 8.2	37.1 ± 4.5	29.0 ± 4.1	32.7 ± 4.6
% S	30.8 ± 1.1	57.5 ± 8.1 *	51.5 ± 7.2 *	34.0 ± 4.1	29.5 ± 3.5	44.0 ± 8.2 **	47.6 ± 9.1 **
% G_2_M	1.74 ± 10.1	16.3 ± 1.9 *	9.1 ± 1.3 *	6.8 ± 0.8 *	31.8 ± 4.8 ***	22.5 ± 3.1 ***	15.8 ± 1.9 ***
%G_2_M/G_0_-G_1_	2.6 ± 0.2	62.5 ± 8.8 **	23.4 ± 2.8 *	11.6 ± 1.7 *	85.7 ± 16.3 ***	77.6 ± 10.8 ***	48.3 ± 6.8 **

Data represent mean ± SEM, *n* = 3. The significance in *p*-values for comparison with control untreated cells is indicated as * *p* < 0.05, ** *p* < 0.01, *** *p* < 0.001.

## Data Availability

The data presented in this study are available in this article.

## Supplementary Materials

The following are available online, Figure S1. Dose-response curves for the antiproliferation assay showing the effects of EC19, EC23 and ATRA on the viability of different cancer and normal cell lines; Table S1. The sequences for primers used in quantitative real time PCR (qPCR) for gene expression analysis.

## Sample Availability

Samples of the compounds are not available from the authors.
