# Supplementary material for: Revealing the Potential Application of EC-Synthetic Retinoid Analogues in Anticancer Therapy
Source: Molecules. 2021 Jan 19;26(2):506. doi: 10.3390/molecules26020506 (PMC7835894; doi:10.3390/molecules26020506)
Supplement: Supplementary file 1 [file molecules-26-00506-s001.pdf]

Revealing the potential application of EC-synthetic retinoid analogues in anticancer therapy

Mohamed R. Abdelaal[1,2], Sameh H. Soror[1,2], Mohamed R. Elnagar[3] and Hesham Haffez[1,2,*]

[1]Biochemistry and Molecular Biology Department, Faculty of Pharmacy, Helwan University, Cairo 11795, Egypt; mohamed_abdelaal@pharm.helwan.edu.eg (M.R.A.); sameh_soror@pharm.helwan.edu.eg (S.H.S.)

[2]Center of Scientific Excellence "Helwan Structural Biology Research, (HSBR)", Helwan University, Cairo 11795, Egypt

[3]Department of Pharmacology and Toxicology, Faculty of Pharmacy, Al-Azhar University, Cairo 11823, Egypt; mohamed.r.elnagar@azhar.edu.eg (M.R.E)

***Correspondence: hesham.haffez@pharm.helwan.edu.eg (H.H.); Tel.: +20-1094970173

Table S1. The sequences for primers used in quantitative Real Time Reverse-Transcriptase PCR (RT-qPCR). Page (2)

Figure S1. The effects of EC19, EC23 and ATRA on the viability of different cancer and normal cell lines. Page (3)

Supplementary Materials

Table S1. The sequences for primers used in quantitative real time PCR (qPCR) for gene expression analysis.

Gene	Primer sequence
**RARα**	F: 5'- GGGCAAATACACTACGAACAACA -3'<br>R: 5'- CTCCACAGTCTTAATGATGCACT -3'
**RARβ**	F: 5'- TCGGCACACTGCTCAATC -3'<br>R: 5'- GAAGCAGGGTTTGTACACTCG -3'
**RARγ**	F: 5'- CTGGAGATGGATGACACC -3'<br>R: 5'- GTTCTCCAGCATCTCTCG -3'
**Caspase 3**	F: 5'- ACATGGAAGCGAATCAATGGACTC -3'<br>R: 5'- AAGGACTCAAATTCTGTTGCCACC -3'
**Caspase 8**	F: 5'- AGAGTCTGTGCCCAAATCAAC -3'<br>R: 5'- GCTGCTTCTCTCTTTGCTGAA -3'
**Cytochrome C**	F: 5'- GAGGCAAGCATAAGACTGGA -3'<br>R: 5'- TACTCCATCAGGGTATCCTC -3'
**WRN**	F: 5'- GCATGTGTTCGGAAGAGTGTTT -3'<br>R: 5'- TGACATGGAAGAAACGTGGAA -3'
**RAI2**	F: 5'-CGGTCATTAAGATGGGAAGTGAG-3'<br>R: 5'- GAGGCTCGGATTTCCGGTG-3'
**Bax**	F: 5'- CCCGAGAGGTCTTTTTCCGAG -3'<br>R: 5'- CCAGCCCATGATGGTTCTGAT -3'
**BCL-2**	F: 5'- TTGTGGCCTTCTTTGAGTTCGGTG -3'<br>R: 5'- GGTGCCGGTTCAGGTACTCAGTCA -3'
**p53**	F: 5'- GCCCAACAACACCAGCTCCT -3'<br>R: 5'- CCTGGGCATCCTTGAGTTCC -3'
**E-cadherin 1**	F: 5'- ATTTTTCCCTCGACACCCGAT -3'<br>R: 5'- TCCCAGGCGTAGACCAAGA -3'

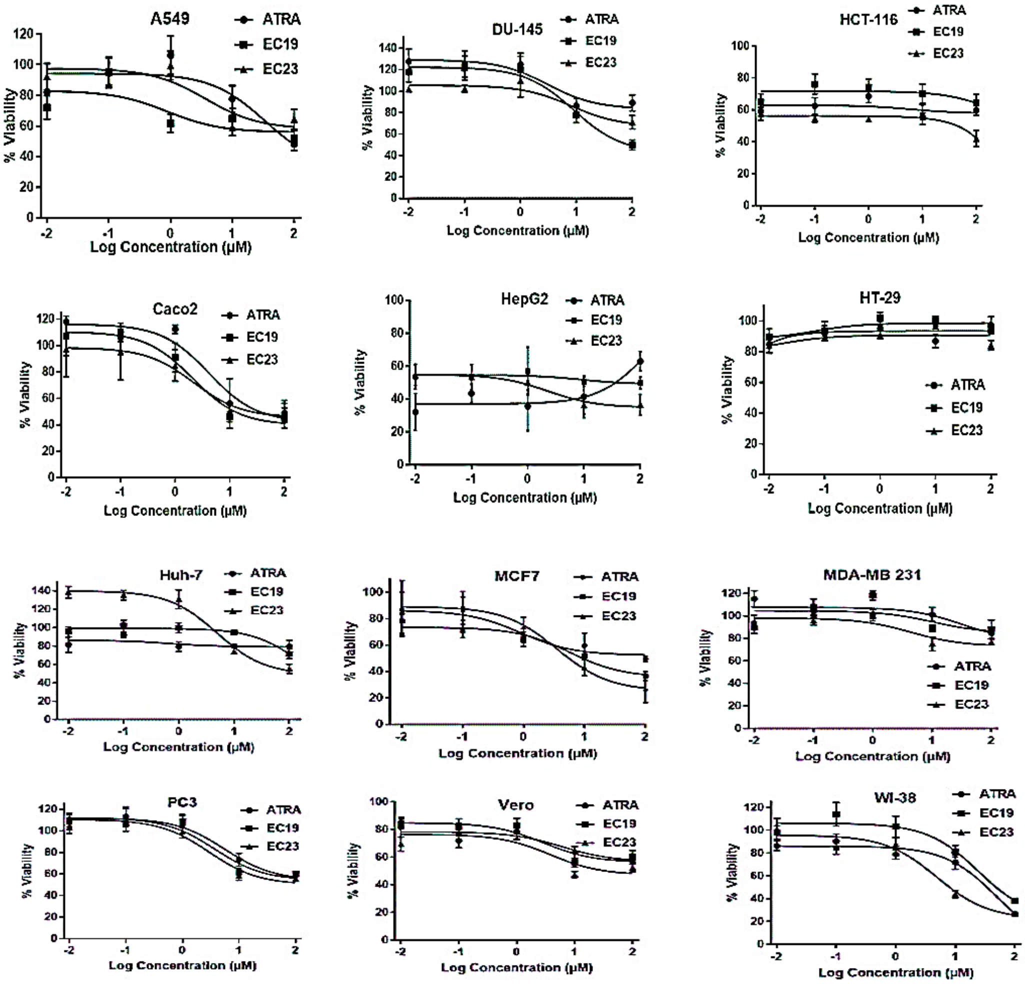

Figure S1. Dose-response curves for the antiproliferation assay showing the effect of EC19, EC23 and ATRA on the viability of different cancer and normal cell lines. Cells were treated with the different serial concentrations. The antiproliferation and cytotoxicity was assessed by MTT assay. Values are presented as mean ± SEM of three independent experiments.
